# Therapeutic Plasma Exchange in Severe Rhabdomyolysis: A Case-Control Study

**DOI:** 10.7759/cureus.39748

**Published:** 2023-05-30

**Authors:** Sukhmani Boparai, Rachaita Lakra, Lovekirat Dhaliwal, Rajkamal S Hansra, Mohammad Alfrad Nobel Bhuiyan, Steven A Conrad, Prathik Krishnan

**Affiliations:** 1 Internal Medicine, Louisiana State University (LSU) Health Shreveport, Shreveport, USA; 2 Pulmonary and Critical Care Medicine, Louisiana State University (LSU) Health Shreveport, Shreveport, USA

**Keywords:** non-traumatic rhabdomyolysis, rhabdomyolysis, renal outcomes, plasmapheresis, therapeutic plasma exchange

## Abstract

Introduction: Rhabdomyolysis is a serious condition that can cause acute kidney injury (AKI), compartment syndrome, severe metabolic and electrolyte derangement leading to arrhythmias, and even death. Total plasma exchange (TPE) has been used as a treatment modality to clear myoglobin, but the evidence is limited. In this study, we aim to investigate the use of TPE in critically ill rhabdomyolysis patients.

Methods: We retrospectively chart reviewed adult patients admitted to the intensive care unit (ICU) with a diagnosis of rhabdomyolysis between 2012 and 2021. We dichotomized patients into two groups based on whether TPE was used or not in addition to standard care. PRISMA machines with TPE2000 filters and either 5% albumin or fresh frozen plasma were used in the TPE group.

Results: The patients' age ranged from 23 years to 87 years (mean 49.4, SD 18.1), and 51% were male. Initial creatinine ranged from 0.6 to 16mg/dL (mean 3.4, SD 2.7), creatinine phosphokinase (CPK) from 403-93,232 U/L, and myoglobin from 934 to >20,000. The Sequential Organ Failure Assessment (SOFA)scores on admission ranged from 6 to 17 (mean 7.23, SD 3.40). Overall, 28.78% (N=19) of the patients received therapeutic plasma exchange. The overall mortality in our study was 31.9%, with the length of ICU stay ranging from 1-25 days (mean 7.10, SD 5.91) among survivors. Older age and the presence of shock were predictive of mortality in univariate and multivariate analyses. There was no statistically significant association in mortality between the TPE and non-TPE groups (36.84% in TPE vs. 36.17% in the non-TPE group, OR 0.7209, p=0.959). Only two patients in the non-TPE group developed CKD/ESRD on long-term follow-up.

Conclusion: Our study showed that TPE administration in critically ill patients with rhabdomyolysis did not improve mortality or length of ICU stay. Further studies are required to elucidate its indication and effect on long-term renal outcomes.

## Introduction

Rhabdomyolysis is a syndrome caused by direct or indirect injury to skeletal muscle and the release of its breakdown products into circulation. It can be asymptomatic or progress to acute kidney injury (AKI), compartment syndrome, severe metabolic and electrolyte derangement leading to arrhythmias, and even death. The incidence of rhabdomyolysis in critically ill patients has been reported to range from 0.09% to 20.4% in various studies [1,2]. ICU patients have many risk factors for developing rhabdomyolyses, such as sepsis, heat stroke, hypothermia, vascular obstruction, prolonged surgery, substance use, and anesthesia. It is often associated with multi-organ dysfunction, most commonly renal failure in up to 65% of patients. Renal dysfunction is mediated by the nephrotoxic effects of myoglobin via multiple mechanisms, including tubular obstruction, direct and ischemic tubular injury, and intrarenal vasoconstriction [3]. Creatine kinase (CK) levels >773 U/L, serum myoglobin >368µg/L, and urine myoglobin > 38 µg/L were shown to predict AKI in one study, with serum myoglobin having the most predictive value [1]. Rhabdomyolysis has a reported mortality of up to 59% in the presence of renal dysfunction.

Treatment of rhabdomyolysis consists of volume expansion with intravenous fluids, renal replacement therapy, and supportive care. Other treatments have been attempted, including urinary alkalinization, forced diuresis, and steroids, but there is insufficient evidence for their benefit [4].

Myoglobin and inflammatory cytokines are believed to play a central role in the pathogenesis of rhabdomyolysis. Due to this, treatments focused on myoglobin removal have been attempted in addition to supportive care. Myoglobin has a molecular mass of 17 kDa and is a non-spherical, electrically charged molecule with a low diffusion coefficient making its removal technically challenging. Current polymeric membranes are ineffective, and even high-flux membranes have a low sieving coefficient for myoglobin. Therapeutic plasma exchange or plasmapheresis membranes have a higher sieving coefficient, but the low volume exchange limits the total clearance. Other approaches that have been used include high-cut-off membranes, direct adsorption on whole blood, or coupled plasma-filtration adsorption [5].

Current guidelines from the American Apheresis Society do not include rhabdomyolysis as an indication for the use of apheresis techniques such as total plasma exchange (TPE); however, many case series have attempted to further explore this question [6]. There is currently limited evidence for the clinical utility of using various extracorporeal therapies for myoglobin clearance. This study shares our institutional experience of using therapeutic plasma exchange in critically ill patients with rhabdomyolysis.

## Materials and methods

This is a retrospective, observational study conducted at an academic tertiary care hospital. We included all patients between the ages of 18-90 years who were admitted to the intensive care unit (ICU) with a diagnosis of rhabdomyolysis or who developed rhabdomyolysis while in the ICU from 2012 to 2021. Patients with incomplete data or missing variables of interest were excluded. The Institutional Review Board approved the study (STUDY00001606), and the Research Ethics Committee waived the requirement for informed consent. This study was not sponsored, and the authors do not have any conflict of interest to report.

We reviewed electronic medical records (Epic, Verona, WI) for age, sex, etiology of rhabdomyolysis, illness severity score (SOFA), lab values including serum chemistries, CK, myoglobin, creatinine before and after treatment, treatment given, timing, and duration of treatments like therapeutic plasma exchange and continuous renal replacement, outcomes like mortality, length of ICU stay, complications of TPE, need for intermittent hemodialysis and long-term renal effects. Data was collected using a secure HIPAA-compliant Excel worksheet.

TPE was used in addition to standard care at the discretion of the treating team. There is limited data on the role of TPE in rhabdomyolysis and considerable variation in practice patterns among attending physicians at our institute. As a result of this, the decision to use TPE was guided by the clinical judgment of the ICU team.

TPE was performed using the Prismaflex and Prismax systems (Baxter International, Deerfield, IL) with the TPE2000 filter, a large-pore, polypropylene membrane plasma filter. Either 5% albumin or fresh frozen plasma was used to perform plasmapheresis or plasma exchange, respectively, at a 25% filtration fraction.

Statistical analysis

We summarized data as mean (standard deviation) for quantitative variables and proportions (%) for discrete variables. Proportions were compared using the Chi-square test for categorical variables of interest. For continuous variables of interest, the t-test was used to compare means.

Univariate analysis was performed by logistic regression to assess the association of variables of interest with the outcome. The primary outcome was mortality, and secondary outcomes included length of ICU stay and long-term renal dysfunction. We then created a multivariate logistic regression model including all variables that reached statistical significance (P < 0.05) on univariate analysis to analyze the effect of multiple factors on outcomes. All analyses were done by using SPSS (ver 28). A P-value less than .05 was considered statistically significant.

## Results

We identified 66 critically-ill patients with rhabdomyolysis. 77.3 % (n=51) of the patients were male with ages ranging from 23 years to 87 years (mean 49.4, SD 18.1 ). 81.8% (n=54) of patients had one or more co-morbidities, with hypertension, diabetes mellitus, and seizure disorder being the most common.

The most common etiologies for rhabdomyolysis included substance use (24.6%), sepsis (13%), seizures (13%), trauma (8.7%), and limb ischemia (8.9%). Initial creatinine ranged from 0.6-16 (mean 3.4, SD 2.7), creatinine phosphokinase (CPK) from 403 to 93,232 U/L, and myoglobin from 934 to >20,000. SOFA scores on admission ranged from 6 to 17 (mean 7.27, SD 3.40). 53.03% (n=35) of patients had shock requiring vasopressors, 74.24% (n=49) had respiratory failure requiring mechanical ventilation, and 31.81% (21) had renal failure requiring continuous renal replacement via hemofiltration. 28.78% (n=19) of the patients received plasmapheresis or therapeutic plasma exchange with full FFP or FFP/albumin replacement. TPE was started within 48 hours of diagnosis in all patients.

The overall mortality in our study was 36.36% (n=24), with the length of ICU stay ranging from one to 25 days (mean 9.05, SD 9.09) among survivors. In addition, 15.9% (n=11) of patients required intermittent hemodialysis (IHD) during the hospital stay, with 4.3% requiring continued IHD at discharge (Table 1).

**Table 1:** Baseline characteristics and outcomes Cr - serum creatinine, SOFA - Sequential Organ Failure Assessment

Patient characteristics	No TPE used, (N=47, 71.21%)	TPE used (N=19, 28.78%)	Total (N=66, 100%)
Age in years, mean (SD)	52.19 (18.544)	42.47 (15.39)	49.39 (18.13)
Sex, % males (n)	74.46% (35)	84.21% (16)	77.3% (51)
Initial Cr in mg/dl, mean (SD)	3.26 (3.0)	3.60 (1.60)	3.36 (2.70)
SOFA score, mean (SD)	6.59 (3.47)	9.0 (2.71)	7.27 (3.43)
Shock present, %	55.32% (26)	47.37% (9)	53.03% (35)
Intubated, %	74.47% (35)	73.68% (14)	74.24% (49)
Mortality, %	36.17% (17)	36.84% (7)	36.36% (24)
Length of ICU stay in days, mean (SD)	8.89 (9.92)	9.53 (6.02)	9.05 (9.09)

On multivariate regression analysis, older age (OR 0.961, p=0.035) and presence of shock were (OR 0.21, p=0.04) predictive of mortality but not sex, mechanical ventilation, SOFA scores, CRRT, or TPE (Table 2).

**Table 2:** Predictors of mortality Cr-Serum creatinine, SOFA-Sequential Organ Failure Assessment

Patient characteristics	P-value	Multivariate odds ratio (95% CI)
Age (unit odds ratio )	0.024	0.956 (0.919-0.994)
Sex :		0.833 (0.182-3.816)
Female	REF	
Male	0.813	
Initial Cr (unit odds ratio)	0.187	0.780 (0.539-1.129)
SOFA score (unit odds ratio)	0.530	1.094 (0.827-1.447)
Presence of shock:		0.212 (0.045-0.987)
Yes	REF	
No	0.048	
Intubation:		0.138 (0.019-1.1015)
Yes	REF	
No	0.052	
TPE use:		1.02 (0.036-2.85)
No	REF	
Yes	0.959	

The patients who received TPE were younger (42.47 years vs. 52.19 years, p=0.048) and had a similar gender distribution (84.21% males in TPE vs. 74.46 % in the non-TPE group, p=0.558). Both groups had similar mean creatinine on admission (3.60 vs. 3.21, p-value 0.6125) but the TPE group had a higher SOFA score (9 vs. 6.45, p=0.008). In the TPE group, all patients required CRRT vs. only 6.38 % in the non-TPE group (p<0.001). 74.47% in TPE were intubated vs 73.68% (p=0.992) in the non-TPE, and 47.4% of patients were on vasopressors in the TPE vs 55.3% in the non-TPE group (p=0.558).

There was no statistically significant difference in mortality between the two groups (36.17 % in the non-TPE group vs 36.84% in TPE group, OR 1.02, p=0.959) or length of ICU stay (9.53 in TPE vs. 8.89 in the non-TPE group, p=0.349). Two patients in the non-TPE and one patient in the TPE group required continued intermittent hemodialysis on hospital discharge. Out of these, both the patients in the non-TPE group developed CKD/ESRD on long-term follow-up, and the patient in the TPE group had complete renal recovery at three months. No complications of TPE were reported in this study.

## Discussion

We show that therapeutic plasma exchange can be safely used for myoglobin clearance with critically ill patients with severe rhabdomyolysis. Although no difference in mortality or length of stay was seen in the two groups, none of the patients who received TPE developed long-term renal dysfunction. There is limited data on using therapies targeting myoglobin and other large molecule clearance in severe rhabdomyolysis.

We reviewed 15 case reports and case series published between 2000-2021 that attempted myoglobin clearance in a total of 27 critically ill patients with rhabdomyolysis. The age ranged from 15 to 78 years and was predominantly male. The etiology of rhabdomyolysis included sepsis, polytrauma, necrotizing fasciitis, heatstroke, PRIS, snakebite, and mushroom poisoning and all patients had multi-organ dysfunction, including renal dysfunction requiring continuous renal replacement therapy. Various modalities were used for myoglobin clearance, with the most common being plasmapheresis/therapeutic plasma exchange (nine studies), coupled plasma filtration-absorption (1 study, n=4; Pezzi et al.), high flux dialyzer (1 study, n=6; Sorrenti), super high flux membrane (n=1; Naka), and the Cytosorb system, which is a hemoadsorption cartridge added to the hemodialysis circuit (three studies, n=3) [7-17]. On the other hand, several case reports and case series published during this time did not use any modality for myoglobin clearance and used supportive care and other treatments [18-28].

It is difficult to generalize these case reports/series results due to their small size, heterogeneity inpatient population, geographical location, inconsistent reporting of illness severity scores, and other treatments. In addition, the details for equipment and techniques used for myoglobin removal and outcome measures like the length of stay and long-term renal dysfunction are also inconsistently reported.

A few more extensive studies have evaluated the utility of extracorporeal therapies for severe rhabdomyolysis. A randomized control trial enrolling 70 critically ill patients with rhabdomyolysis requiring renal replacement therapy evaluated the efficacy of using a high-cutoff dialyzer in a continuous venovenous circuit. Myoglobin clearance was significantly higher in the high cutoff dialyzer arm than in the control arm used CVVHDF with a high-flux dialyzer. However, ICU mortality was higher in the intervention arm, but there were no differences in hospital, 28- and 90-day mortality [29].

Another retrospective study used the Cytosorb system, a cytokine adsorption filter, in 43 ICU patients with rhabdomyolysis. This study also showed a significant reduction of myoglobin during Cytosorb treatment but did not comment on other clinical outcomes like mortality, renal recovery, or length of stay [30].

These studies highlight the gaps in our current understanding of the clinical utility of extracorporeal therapies in rhabdomyolysis. In addition, our study adds to the limited data on this subject. The main strength of our study is its size, focus on clinical outcomes and long-term follow-up of patients.

We acknowledge several limitations of our study, most notably its retrospective design, lack of randomization or matching, and variability in practice patterns and threshold for initiating TPE between different providers due to the lack of institutional treatment protocols for initiating TPE. Since the cases were retrieved using billing/diagnostic codes and relied heavily on appropriate documentation in the electronic health record, it is possible to have missed some eligible patients, which could have affected results given the small sample size. Also, the complications of TPE may be underreported due to missing documentation. We could not comment on myoglobin clearance as it was not measured as part of routine care.

## Conclusions

Our study did not show a mortality benefit or a difference in the length of ICU stay in patients receiving TPE for severe rhabdomyolysis. At the same time, TPE did not confer a statistically significant decrease in long-term dialysis needs. However, our study had limitations of having a small sample size and heterogeneity of treatment initiation times. Further studies are needed to determine if TPE has a role in preventing long-term renal dysfunction.

## References

[1] El-Abdellati E, Eyselbergs M, Sirimsi H, Hoof VV, Wouters K, Verbrugghe W, Jorens PG (2013). An observational study on rhabdomyolysis in the intensive care unit. Exploring its risk factors and main complication: acute kidney injury. Ann Intensive Care.

[2] de Meijer AR, Fikkers BG, de Keijzer MH, van Engelen BG, Drenth JP (2003). Serum creatine kinase as predictor of clinical course in rhabdomyolysis: a 5-year intensive care survey. Intensive Care Med.

[3] Williams J, Thorpe C (2014). Rhabdomyolysis, continuing education in Anaesthesia Critical Care & Pain. BJA Edu.

[4] Zhang L, Kang Y, Fu P (2012). Myoglobin clearance by continuous venous-venous haemofiltration in rhabdomyolysis with acute kidney injury: a case series. Injury.

[5] Ronco C (2005). Extracorporeal therapies in acute rhabdomyolysis and myoglobin clearance. Crit Care.

[6] Padmanabhan A, Connelly-Smith L, Aqui N (2019). Guidelines on the use of therapeutic apheresis in clinical practice - evidence-based approach from the writing committee of the American Society for Apheresis: the eighth special issue. J Clin Apher.

[7] Scharf C, Liebchen U, Paal M, Irlbeck M, Zoller M, Schroeder I (2021). Blood purification with a cytokine adsorber for the elimination of myoglobin in critically ill patients with severe rhabdomyolysis. Crit Care.

[8] Le HQ, Nguyen NT, Vo TN (2021). Envenoming by king cobras (Ophiophagus hannah) in Vietnam with cardiac complications and necrotizing fasciitis. Toxicon.

[9] Colaco CM, Basile K, Draper J, Ferguson PE (2021). Fulminant Bacillus cereus food poisoning with fatal multi-organ failure. BMJ Case Rep.

[10] Miyake T, Okada H, Kanda N (2020). Multiple trauma including pelvic fracture with multiple arterial embolization: an autopsy case report. Thromb J.

[11] Dilken O, Ince C, van der Hoven B, Thijsse S, Ormskerk P, de Geus HR (2020). Successful reduction of creatine kinase and myoglobin levels in severe rhabdomyolysis using extracorporeal blood purification (CytoSorb®). Blood Purif.

[12] Lu Z, Chen YB, Huang B, Peng S, Wang QW, Liu DL, Wang H (2018). Mixed amanita phalloides poisoning with rhabdomyolysis: analysis of 4 cases (Article in Chinese). Nan Fang Yi Ke Da Xue Xue Bao.

[13] Gong PH, Dong XS, Li C (2017). Acute severe asthma with thyroid crisis and myasthenia: a case report and literature review. Clin Respir J.

[14] Jiao J, Zhou F, Kang H, Liu C, Yang M, Hu J (2017). Unexpected extrapyramidal symptoms and pulmonary aspergillosis in exertional heatstroke with fulminant liver failure: a case report. J Med Case Rep.

[15] Mita K, Tsugita K, Yasuda Y (2017). A successfully treated case of cardiac arrest after Caesarean section complicated by pheochromocytoma crisis and amniotic fluid embolism. J Anesth.

[16] Yoshizawa T, Omori K, Takeuchi I (2016). Heat stroke with bimodal rhabdomyolysis: a case report and review of the literature. J Intensive Care.

[17] Levin PD, Levin V, Weissman C, Sprung CL, Rund D (2015). Therapeutic plasma exchange as treatment for propofol infusion syndrome. J Clin Apher.

[18] Sorrentino SA, Kielstein JT, Lukasz A, Sorrentino JN, Gohrbandt B, Haller H, Schmidt BM (2011). High permeability dialysis membrane allows effective removal of myoglobin in acute kidney injury resulting from rhabdomyolysis. Crit Care Med.

[19] Kasugai D, Tajima K, Jingushi N, Uenishi N, Hirakawa A (2020). Multiple limb compartment syndrome as a manifestation of capillary leak syndrome secondary to metformin and dipeptidyl peptidase IV inhibitor overdose: a case report. Medicine (Baltimore).

[20] Esposito P, Estienne L, Serpieri N (2018). Rhabdomyolysis-associated acute kidney injury. Am J Kidney Dis.

[21] Shibamori K, Yamamoto T, Matsuki M, Matsuda Y, Kato S, Takei F, Yanase M (2014). Rhabdomyolysis after radical nephrectomy in the lateral decubitus position: report of 2 cases (Article in Japanese). Nihon Hinyokika Gakkai Zasshi.

[22] Fang S, Xu H, Zhu Y, Jiang H (2013). Continuous veno-venous hemofiltration for massive rhabdomyolysis after malignant hyperthermia: report of 2 cases. Anesth Prog.

[23] Rhidian R (2011). Running a risk? Sport supplement toxicity with ephedrine in an amateur marathon runner, with subsequent rhabdomyolysis. BMJ Case Rep.

[24] Zhou F, Song Q, Peng Z (2011). Effects of continuous venous-venous hemofiltration on heat stroke patients: a retrospective study. J Trauma.

[25] Shah SV, Reddy K (2010). Rhabdomyolysis with acute renal failure triggered by the seasonal flu vaccination in a patient taking simvastatin. BMJ Case Rep.

[26] Kutlesa M, Lepur D, Bukovski S, Lepur NK, Barsić B (2009). Listeria monocytogenes meningitis associated with rhabdomyolysis and acute renal failure. Neurocrit Care.

[27] Inoue S, Nagayama M, Aoki H (2007). Continuous venovenous hemodiafiltration for life-threatening mitochondrial myopathy with lactic acidosis and rhabdomyolysis. J Intensive Care Med.

[28] Galea M, Jelacin N, Bramham K, White I (2007). Severe lactic acidosis and rhabdomyolysis following metformin and ramipril overdose. Br J Anaesth.

[29] Splendiani G, Mazzarella V, Cipriani S, Pollicita S, Rodio F, Casciani CU (2001). Dialytic treatment of rhabdomyolysis-induced acute renal failure: our experience. Ren Fail.

[30] Weidhase L, de Fallois J, Haußig E, Kaiser T, Mende M, Petros S (2020). Myoglobin clearance with continuous veno-venous hemodialysis using high cutoff dialyzer versus continuous veno-venous hemodiafiltration using high-flux dialyzer: a prospective randomized controlled trial. Crit Care.

